# Pharmacist-Led Antimicrobial Stewardship Programme in Two Tertiary Hospitals in Malawi

**DOI:** 10.3390/antibiotics13060480

**Published:** 2024-05-23

**Authors:** Nelson Nyoloka, Charlotte Richards, William Mpute, Hope Michael Chadwala, Hanna Stambuli Kumwenda, Violet Mwangonde-Phiri, Aggrey Phiri, Ceri Phillips, Charlotte Makanga

**Affiliations:** 1 Pharmaceutical Society of Malawi, Lilongwe P.O. Box 2240, Malawi; 2 School of Life Sciences and Applied Health Professions, Kamuzu University of Health Sciences, P/Bag 360, Chichiri, Blantyre 3, Malawi; 3 Welsh Antimicrobial Pharmacist Group, Pharmacy Department, Ysbyty Gwynedd, Bangor LL57 2PW, Wales, UK; 4 Pharmacy Department, Morriston Hospital, Swansea Bay University Health Board, Swansea SA6 6NL, Wales, UK; 5 Kamuzu Central Hospital, Lilongwe P.O. Box 149, Malawi; 6 Tidziwe Centre, University of North Carolina Project, Lilongwe P/Bag A-104, Malawi; 7 Mzuzu Central Hospital, Mzuzu Private Bag 209, Malawi; 8 Pharmacy Department, Grange University Hospital, Aneurin Bevan University Health Board, Llanfrechfa NP44 8YN, Wales, UK; 9 Pharmacy Department, Ysbyty Gwynedd, Betsi Cadwaladr University Health Board, Bangor LL57 2PW, Wales, UK

**Keywords:** antimicrobial stewardship, point prevalence survey, education, referral hospital, toolkit, CwPAMS, health partnerships

## Abstract

The ultimate goal of antimicrobial stewardship (AMS) programmes is to decrease the occurrence and spread of antimicrobial resistance (AMR). In response to this, a pharmacist partnership was established between Malawi and Wales (UK) with the aim of strengthening antimicrobial stewardship (AMS) activities in Malawi, with the initial project focusing on two tertiary referral hospitals. The Global Point Prevalence Survey (GPPS) was undertaken for the first time in Malawi at these sites and demonstrated a prescribing rate slightly lower than the African average, with ceftriaxone indicated for almost every bacterial infection. An educational intervention was also delivered, with a train-the-trainer approach upskilling pharmacists at the two sites, who then cascaded co-produced training sessions to an additional 120 multidisciplinary health professionals. A toolkit to support AMS at an individual patient level was also developed and disseminated to provide an ongoing reference to refer to. Both the trainings and toolkit were well received. Over the course of this project, significant progress has been made with the AMS programmes at the two sites, with local staff empowered to implement AMS activities. These interventions could be easily replicated and scaled and support the delivery of some of the AMS elements of the Malawi Ministry of Health National Action Plan for Antimicrobial Resistance.

## 1. Introduction

Global mortality attributed to antimicrobial resistance (AMR) is estimated at 1.27 million deaths yearly [1,2]. AMR is proving to be a public health burden hindering the delivery of healthcare at all levels in healthcare facilities, predominantly in low- and middle-income countries (LMICs) [2,3,4]. In sub-Saharan Africa, it has been estimated that 27.3 deaths per 100,000 are due to AMR and therefore remains the highest AMR mortality rate in the world [2,3,5]. The AMR crisis is projected to cause 300 million cumulative premature deaths by 2050, causing an economic loss of up to $100 trillion (£64 trillion) globally [1,6,7].

The World Health Organisation (WHO) and many other nongovernmental organisations (NGOs) have raised awareness of AMR through different platforms [1,4,8,9,10]. While some high-income countries have prioritised the AMR fight [7,11], LMICs face challenges in the fight against AMR.

Globally, antimicrobial misuse and overuse propagates the development and escalation of AMR [1,4,8,9,11,12,13,14]. Optimising the use of antimicrobial agents is therefore a key element of the global response to the AMR crisis [2,3,4,9,11]. Many countries are adopting the concept of antimicrobial stewardship (AMS) at national and organisational levels [2,4,7,8,15,16,17,18,19]. Implementation of AMS activities in LMICs has proved to be challenging due to a high infectious disease burden, limited access to some useful antibiotics, unregulated use of antibiotics in the community, and limited diagnostic capacity to guide clinical decision-making [19,20,21,22]. In Malawi, there is an urgent need to set up locally adapted, sustainable, and scalable interventions to contain the problem of AMR. Responding to this need, the Republic of Malawi developed a National Action Plan (NAP) for Antimicrobial Resistance Strategy (2017–2022) [23].

In response to the Malawi NAP and worldwide need to fight against AMR [4,11,12,23,24], volunteers from the Pharmaceutical Society of Malawi (PHASOM) and the Welsh Antimicrobial Pharmacy Group (WAPG) formed the Malawi-Wales Antimicrobial Pharmacy Partnership. In 2021, the partnership secured a Commonwealth Partnerships for Antimicrobial Stewardship (CwPAMS) grant to work with two referral hospitals in Malawi, Kamuzu Central Hospital (KCH) and Mzuzu Central Hospital (MCH), located in the central and northern regions and caring for almost 7.5 and 2.3 million Malawians, respectively [25]. The aim of the project was to support and expand AMS activities, which were in their infancy within the two hospitals. This included introducing a regular point prevalence survey (PPS) to establish baseline antimicrobial prescribing practices and monitor change, the development of a practical AMS toolkit to support AMS at a patient level, and multidisciplinary training sessions to build capacity and support the delivery of a robust and sustainable AMS programme within KCH and MCH.

## 2. Materials and Methods

### 2.1. Study Design

The PHASOM and WAPG collaborated on the study design and submitted an application for CwPAMS funding in the summer of 2021. Following the successful grant approval in the autumn of 2021, the partnership progressed detailed plans for the delivery of the project, which included three main targets to support AMS in Malawi. These were the implementation of robust and sustainable antimicrobial surveillance utilising the Global Point Prevalence Survey (GPPS), development of a practical toolkit to support AMS at an individual patient level, and a multidisciplinary educational training session on AMR and AMS. The partnership had weekly virtual meetings and regular email communication over the course of the project, to monitor progress against agreed actions, develop and organise delivery of the training sessions and AMS toolkit, and support the development of local AMS programmes.

Initially, the WAPG team planned to visit Malawi in November 2022, to coincide with the baseline GPPS data collection. However, due to COVID-19 travel restrictions and increased work pressures at hospital level, this was not possible, and the WAPG team visit was postponed until February 2022. Thus, completion of the baseline GPPS was postponed until January 2022 and the WAPG team provided support and training virtually as required.

During the visit, the WAPG team visited the study sites to observe clinical practice and procedures and met with key local stakeholders, such as medical directors or AMS committee leads. The team also met with the Fleming Fund country grant holders, to ensure the work of the project dovetailed with related work being undertaken to improve microbiology capacity and AMS capabilities. Individuals involved in the Fleming Fund country grant also shared their training on AMS to ensure collaboration and avoid duplication. GPPS data were interrogated by the WAPG team to identify potential target areas, which were discussed with the local AMS leads. A virtual scoping exercise was undertaken prior to the visit to map the infection management pathway, which was later refined during the visit.

### 2.2. Global Point Prevalence Survey of Antimicrobial Consumption and Resistance (GPPS)

Baseline prescribing practices were established utilising GPPS methodology [26] over a two-week period in January 2022. Data were collected from prescription charts for all inpatients by a multidisciplinary team with the support of the AMS lead at each site. There was a mixture of paper-based and electronic data collection across the two sites depending on the availability of devices and Internet connection. Anonymised data were entered, validated, and reported using the GPPS web platform. GPPS training and support with data validation was provided remotely by the WAPG team members.

### 2.3. Development of Training Package

A team approach was taken to the development of the training tools. This was performed using several information sources, to ensure it was fit for purpose for the prevailing healthcare settings. Themes from the GPPS data were identified to ensure that the content of the training was relevant. Initial training with the three AMS lead pharmacists was completed from the tertiary hospitals, in February 2022, one from MCH and two from KCH, during which AMR and AMS themes were covered. This formed the start of the train-the-trainer model used throughout the project. These pharmacists then formed the working group to develop the training. The working group divided into 3 themes:

1: Introduction to antimicrobial resistance;
2: All about antibiotics;
3: Antimicrobial stewardship and putting this into action.

The training was co-developed and written by the working group of pharmacists from Malawi and Wales. Once written, the training was reviewed by the wider team, including the Fleming Fund country grant holders, which was key to successful implementation. The training used available resources such as The Antimicrobial Stewardship Game (an interactive board game by Focus Games™) and case studies. The full timetable for the day was planned by the team and invitations to attend training extended to all healthcare professionals. The training was run on each site over two days and also included training on sample collection, microbiology, and infection prevention and control; colleagues from infection prevention and control and the microbiology labs supported these latter aspects of the training. An overview of the training sessions is available in Supplementary Materials S2.

The training was evaluated by the participants pre- and post-training using both an online and paper questionnaire, for those unable to access the online survey (Supplementary Materials). The questionnaire was adapted from a tried-and-tested model from other CwPAMS projects. This evaluated both the learning of the participants and the quality of the training.

### 2.4. Toolkit Development

A clinical toolkit to support the rational use of antibiotics at a patient level was developed (Supplementary Materials S2). The toolkit included AMS guidance and rationale and links to resources to support clinical decision-making for individual patients. Content was tailored to practice in Malawi, incorporating only agents on the Malawi Essential Medicines List. Malawian resources and evidence were utilised wherever possible and if unavailable, African or international resources were referenced in that order. Links to medicines-related resources were selected that were open access, evidence-based, and regularly updated. As separate Malawi National Treatment Guidelines were already available, these were signposted, but not included in the toolkit. A draft outline for the toolkit was developed and agreed with the AMS pharmacy leads in the two hospitals. The draft toolkit was checked for clinical accuracy by the WAPG pharmacists and reviewed by three pharmacists with hospital-based experience in Malawi to ensure applicability.

The 34-page toolkit was referenced in the training sessions and provided as a take-away resource that participants could use to examine topics in more depth. Paper copies were provided as the mobile data required to download an electronic version were prohibitive for some staff. The toolkit was evaluated with dedicated questions as part of the training questionnaires, distributed immediately post-training.

## 3. Results

### 3.1. Antimicrobial Use and Prevalence

Baseline overall prevalence of antibiotic use was 45% and 40.5% for KCH and MCH, respectively. Prescribing prevalence, summarised in Table 1, ranged from 16.7% to 87% depending on patient population and clinical speciality. Intravenous (IV) therapy accounted for >92% of all prescriptions included in the baseline GPPS.

Ceftriaxone was the most commonly prescribed antibiotic in both hospitals, accounting for 42% and 40% of the antibacterial prescriptions for KCH and MCH, respectively. Furthermore, ceftriaxone was the most commonly prescribed antibiotic outside of the guidelines. Community-acquired infections accounted for >90% of therapeutic antimicrobial use, with pneumonia or lower respiratory tract infections being the most common diagnosis. At baseline, there was no targeted therapy based on the microbiology data in MCH and only two patients received culture-guided treatment in KCH during the survey period.

Overall quality indicators for antimicrobial use were good across the majority of specialities compared with the benchmark level for African hospitals in the GPPS (Table 2). However, KCH ICU had the lowest percentage of documented indication and stop/review dates, though the number of patients surveyed in this unit were small. A small number of prescriptions could not be assessed for guideline compliance as guidelines were not available for these indications.

Results from the baseline GPPS were fed back to local AMS committees during an in-country visit by WAPG members, and target areas for improvement were identified for incorporation into the AMS toolkit and educational sessions. These included more in-depth review of the management of community-acquired pneumonia, promoting IV to oral switch, increasing microbiology sample collection and utilisation of microbiology sample results to guide therapy, and increasing awareness of guidelines.

Following the completion of the education training sessions and introduction of the practical AMS toolkit in May 2022, the GPPS was repeated over a two-week period at both hospitals in June 2022. This was completed following the same process as the baseline GPPS. No significant changes were identified between the two GPPS data sets.

### 3.2. Training

**Figure 1:**
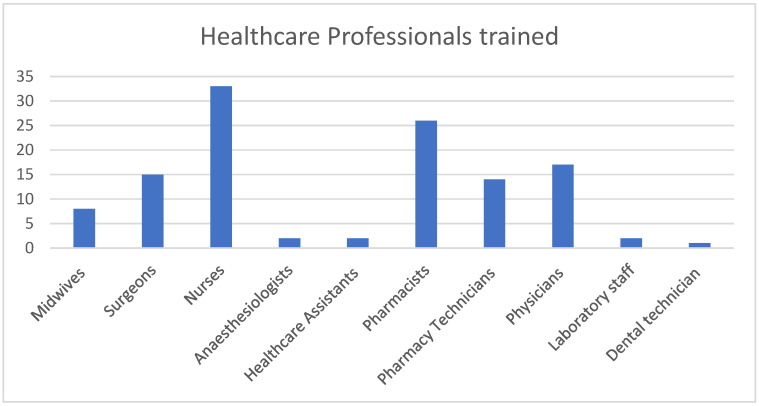
Total number of each healthcare professional group trained.

The training was delivered to 120 healthcare professionals in the two sites by three trained trainers. In total, 60 (50%) were male and 60 (50%) were female, with 10 different healthcare professions represented at the trainings (Figure 1).

**Figure 2:**
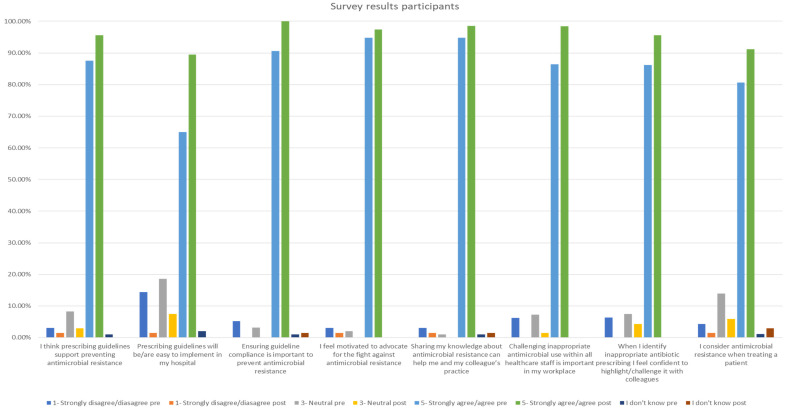
Participant survey results pre- and post-training.

The pre-training questionnaire was completed by 107 participants and the post-training one by 72 participants. The survey results showed improvement post-training in all AMR- and AMS-related questions. All participants agreed or strongly agreed with the statement ‘Ensuring guideline compliance is important to prevent antimicrobial resistance’. Furthermore, the statement ‘I feel motivated to advocate for the fight against antimicrobial resistance’ was supported, with 97.4% of participants agreeing or strongly agreeing, as displayed in Figure 2.

### 3.3. Toolkit

**Figure 3:**
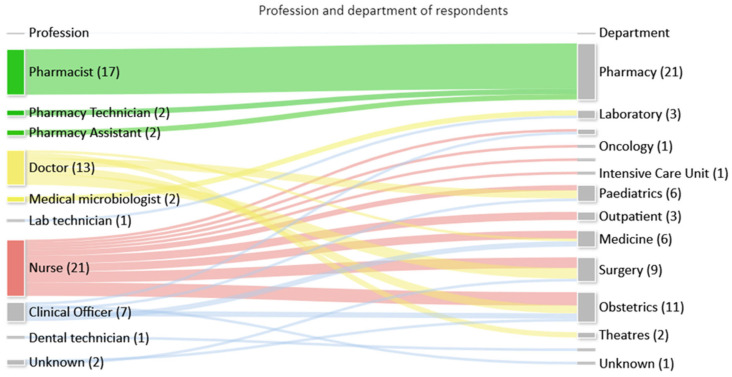
Number of respondents by profession and department.

Feedback on the toolkit was received from 68/120 (57%) multidisciplinary staff that attended training sessions, who represented a range of professions and departments as seen in Figure 3.

Respondents’ experience ranged from 1 month to 30 years (mean 6 years 8 months, median 3 years). About 97% (n = 66) agreed/strongly agreed that the toolkit will support their day-to-day practice, while 96% (n = 68) agreed/strongly agreed that the toolkit was clear and easy to use. Suggestions for improvements were provided by 15 respondents. Not all suggestions were practicable, but potential additions to the toolkit included information on how to safely dispose of antimicrobials and taking a multisectoral approach within the toolkit rather than targeting only the hospital sector.

## 4. Discussion

Establishing working AMS systems in healthcare settings is imperative for the control of AMR. This pharmacy partnership has successfully established a process for monitoring prescribing patterns, developing an educational methodology designed for the issues identified in the two central hospitals and a toolkit to support AMS practice at the patient level. Through the sharing of knowledge, experiences, and resources between the Welsh and Malawian partners, the Malawian pharmacists have been able to lead the introduction of a robust and sustainable AMS programme within their facilities.

Point prevalence surveys are a widely used method to collect information on antimicrobial prescribing practices and other relevant factors in hospitalised patients in LMICs. The GPPS developed to assess both antimicrobial use and AMR allows the identification of opportunities to mitigate the development of AMR [6,8]. It has proved to be a suitable tool for implementing checks and balances on the use of antimicrobials at hospital level and led to the establishment and implementation of AMS programmes in some of the countries that have utilised it [3,5,8,27,28]. Several sub-Saharan African countries have already adopted the use of GPPS as a data collecting tool such as Ghana, Zambia, Uganda, and Tanzania [3,5,27]. Through this project, the GPPS was completed for the first time in Malawi, which provided invaluable insight in prescribing practice. This study demonstrated that GPPS is a simple, feasible, and affordable surveillance method in a resource-limited setting. This information enabled the partnership to highlight potential quality improvement areas to the local AMS committees and support the development of education and training resources. Quarterly GPPS will be conducted at KCH and MCH to ensure ongoing surveillance of antimicrobial use. Furthermore, smaller bespoke GPPS can be employed to monitor progress within specific clinical areas, conditions, or antibiotics to support the local AMS quality improvement programme.

The initial training was delivered using the train-the-trainer approach. Those trained then co-wrote the training to be delivered in the two hospitals, ensuring the content was tailored to the Malawian health institutions. The results from the GPPS also strongly influenced the training material used to ensure that the issues covered were relevant to the healthcare institutions. Working with both the Fleming Fund country grant holders and infection prevention and control teams allowed a multidisciplinary way of working to strongly embed good practice for all. The microbiology sampling training supported by the Fleming Fund country grant team complemented the AMS training sessions, and it is recommended that the two continue to be run alongside each other in future. The train-the-trainer approach is an effective and sustainable way of delivering training, even though staff mobility could affect the sustainability. The materials from the training will support the further implementation and dissemination of information.

Toolkits aimed at the development of AMS programmes at the organisational level have already been published [5,28]; however, they do not provide practical step-by-step clinical AMS advice to frontline healthcare professionals. A literature search failed to find a comprehensive toolkit aimed at optimising antimicrobial use at an individual patient level. Development of the clinical toolkit therefore addresses this gap and provides a standardised approach to AMS at an individual patient level for healthcare professionals in Malawi. It was well received by the vast majority of professionals, regardless of profession, level of experience, and area of clinical practice. Provision of a printed toolkit for each clinical area and making a digital edition available via electronic methods such as QR codes will allow wider, easier, and more cost-effective dissemination. The toolkit could also be adopted by other tertiary hospitals in Malawi or adapted to suit other settings or countries. The feasibility of developing into a single multisectoral resource as suggested by one participant needs to be evaluated, based on observations of other healthcare settings. Clinical pharmacy is in its nascent stages in Malawi. The toolkit will support the delivery of clinical pharmacy in the context of AMS, but the approach could be easily adapted to suit other contexts or clinical areas.

The Malawi AMR National Action Plan includes an ambition to develop structured training materials on optimal antimicrobial use and educate clinicians and other healthcare providers about resistance and optimal prescribing. The combination of training materials and a toolkit provides a free resource that the hospitals can utilise in the future. KCH has already adopted this package as part of their standard educational programme.

## 5. Limitations

The project was time-limited, being only seven months from funding to closure. In addition, progress was hindered by the COVID-19 pandemic; thus, the initial GPPS and the UK team visit were both postponed. This resulted in the training sessions and toolkit being delayed. The post-training GPPS, collected in June 2022, may therefore have been premature as ideally more time would have been allowed following the education sessions to allow the adoption and implementation of AMS practices. The lack of longer-term repeat questionnaires and a longer-term follow-up GPPS are the main limitations of the project and should be undertaken to determine the longer-term impact of these interventions, including the impact of training on the practice and utility of the toolkit in practice.

Ideally, a full co-production approach would have been taken for both the training materials and the toolkit, but this was only feasible for the development of the training materials; however, all elements were reviewed by the Malawi team prior to implementation.

Further research is warranted on AMS activities in Malawi, including the longer-term impact of this project, which will be assessed in a subsequent CwPAMS grant. This project was undertaken in state-funded institutions where treatment is provided free of charge. However, third-sector and private organisations also deliver a significant minority of healthcare in Malawi. Research in both public and privately funded facilities would be useful in the identification of consistencies or variations between them.

## 6. Conclusions

Demonstrating the skills and attributes of pharmacists, particularly as leaders, amongst the multidisciplinary team has been one of the project’s main achievements. Establishing pharmacists as key players in the delivery of the AMS programme will ensure the ongoing engagement and involvement of the profession in providing guidance and advice beyond the project. This will be particularly important for the continuing development and maturity of the local AMS programme in line with the proposed national AMS programme guidelines. Definitely, upscaling AMS activities among medicine custodians in health facilities remains an indispensable approach to tackling AMR.

## Figures and Tables

**Table 1:** Antimicrobial prevalence (%) by continent, hospital, and speciality in January 2022.

	KCH	MCH	Africa GPPS ^a^
**Total**	45	40.5	53.5
**Adults**			
Medical	42.4	38.4	46.7
Surgical	26.2	27.1	51.0
ICU	64.3	0.0	53.2
**Children**			
Medical	56.0	87	73.3
Surgical	45.7	16.7	54.4
ICU	-	-	66.7
**Neonates**			
GNMW	-	64.7	45.8
NICU	-	-	74.6

Abbreviations: ICU: intensive care unit, GNMW: general neonatal medical ward, NICU: neonatal intensive care unit, ^a^ carried out in 39 hospitals.

**Table 2:** Quality indicators for baseline GPPS (January 2022) in two Malawian hospitals (KCH and MCH) compared with Africa benchmark data.

Quality Indicator	KCH % (n)			MCH % (n)		Africa GPPS ^a^ % (n)		
	Medical	Surgical	ICU	Medical	Surgical	Medical	Surgical	ICU
Indication documented	95.1 (290)	82.4 (61)	46.7 (7)	93.8 (61)	100 (26)	66.6 (850)	69.7 (1136)	64 (57)
Guideline compliance *	89.5 (187)	70.4 (38)	55.6 (5)	90 (45)	94.1 (16)	79.4 (405)	72.1 (382)	69.4 (25)
Stop/review date documented	65.9 (201)	78.4 (58)	40 (6)	87.7 (57)	100 (26)	64.6 (825)	72.4 (1180)	60.7 (54)

* Guideline compliance is calculated as compliant to guidelines (yes) when guidelines are existing. ^a^ carried out in 39 hospitals.

## Data Availability

Data available for sharing are contained within the article and Supplementary Materials. Otherwise, some information might be missing deliberately due to ethical policy guidance.

## Supplementary Materials

The following supporting information can be downloaded at: https://www.mdpi.com/article/10.3390/antibiotics13060480/s1; S1: overview of Malawi-Wales Antimicrobial Pharmacy Partnership training sessions; S2: Malawi-Wales Antimicrobial Pharmacy Partnership Antimicrobial Stewardship Toolkit outline, Pre- and post-training questionnaires.
