# Supplementary material for: Pharmacist-Led Antimicrobial Stewardship Programme in Two Tertiary Hospitals in Malawi
Source: Antibiotics (Basel). 2024 May 23;13(6):480. doi: 10.3390/antibiotics13060480 (PMC11201287; doi:10.3390/antibiotics13060480)
Supplement: Supplementary file 1 [file antibiotics-13-00480-s001.zip › Post training survey_Malawi -Wales.pdf]

Dear Colleague:

The Pharmaceutical Society of Malawi (PHASOM) and The Wales Antimicrobial Pharmacist Group (WAPG) applied for funding from the Department of Health and Social Care's Fleming fund, Commonwealth Partnership for antimicrobial stewardship (CwPAMS).

The funding application was successful, and PHASOM and WAPG agreed to develop a Health Partnership with the aim of fostering cooperation and the exchange of knowledge and skills in the areas of: antimicrobial stewardship (AMS) and antimicrobial resistance (AMR).

The purpose of the Partnership is to build professional capacity and sustainability to deliver effective antimicrobial stewardship training to healthcare and pharmacy professionals in both KCH and MCH. This will be namely the education and training of pharmacy teams regarding AMR and AMS strategies, establishing sustainable antimicrobial surveillance systems, improved antimicrobial prescribing and supporting the pharmacy teams to implement AMS strategies.
This survey will help us establish the baseline knowledge, attitudes and behaviours of pharmacists and pharmacy technicians at the KCH and MCH in regards to AMS and AMR.

We ask for the survey to be completed before your the training you are attending and then again after the training so we can understand the learning that we hope will lead to improved AMS practice, and thus lead to tangible benefits for all.

There are no right or wrong answers - you are not being assessed .
If you would like to find out more about the programme, please contact your representative in your hospital in the first instance, and for further queries (and queries directly related to this questionnaire), please get in touch at amr@commonwealthpharmacy.org.

All data collected will be stored anonymously and in line with GDPR best practice (more information can be found here: https://www.gov.uk/data-protection). There is no time limit for these questions and you are free to discontinue at any time. By completing the survey you are confirming that you have read the above and are happy to proceed.

Demographic Questions

1. Name of Partnership (FOR PILOT PHASE ONLY)

- ◯ Norfolk and Suffolk NHS Foundation Trust – The Assemblies of God Hospital, Saboba
- ◯ Malawi Wales Antimicrobial Pharmacist Partnership
- ◯ UK Faculty of Public Health (FPH) – Ghana Public Health Association (GPHA)
- ◯ North Middlesex University Hospital NHS Trust, London (NMUH) – Korle-Bu Teaching Hospital (KBTH)
- ◯ University College London Hospitals NHS Foundation Trust – University of Health and Allied Sciences (UHAS), Ho
- ◯ Healthcare Improvement Scotland – Ghana Police Hospital and Keta Municipal Hospital
- ◯ Northumbria Healthcare NHS Foundation Trust – Kilimanjaro Christian Medical Centre (KCMC)
- ◯ Cambridge University Hospitals NHS Foundation Trust – Makerere University and Mulago National Referral and Teaching Hospital
- ◯ University of Salford – Pharmaceutical Society of Uganda
- ◯ The University of Manchester – Gulu Regional Referral Hospital
- ◯ Nottingham Trent University – Makerere University School of Public Health
- ◯ London School of Hygiene and Tropical Medicine (LSHTM) – Makerere University College of Health Sciences and Infectious Diseases Research Collaboration (IDRC)
- ◯ University of Sussex; Brighton and Sussex Medical School (BSMS) – University Teaching Hospital (UTH), Lusaka

2. Is your hospital a teaching hospital? (i.e. a hospital with structured teaching activities)

- ◯ Yes
- ◯ No

3. What is your main role in the hospital?

- ◯ Medical doctor
- ◯ Pharmacist
- ◯ Nurse
- ◯ Surgeon
- ◯ Medical microbiologist
- ◯ Infectious diseases specialist
- ◯ Infection prevention and control (IPC) specialist
- ◯ Hospital management
- ◯ Other (please specify)

[ ]

4. In which department do you work?

- [ ] Medicine
- [ ] Surgery
- [ ] Paediatrics
- [ ] Obstetrics
- [ ] Accident and emergency
- [ ] Outpatient
- [ ] Other (please specify)

5. How long have you been practicing in this role?

6. We would like to be able to link your answers on this survey with answers on surveys in the future or past. Please enter the code which you entered on the pre-training survey:
- the third letter of your first name
- the month you were born (e.g., if you were born in January you would write “01”)
- the last letter of your mother’s name

Section 0: Perceptions on the Training Toolkit

7. Following your training was the toolkit

	Strongly disagree	Disagree	Neutral	Agree	Strongly agree	I don't know
Clear and easy to use	○	○	○	○	○	○
The toolkit will support my day to day practice	○	○	○	○	○	○

8. What do you think is the most useful part of the toolkit?

9. Do you have any suggestions as to how the toolkit could be improved?

Section 1: Your Views

10. Antibiotics are effective against

- [ ] Bacteria
- [ ] Viruses
- [ ] Fungi
- [ ] Parasites

11. What is antibiotic resistance?

12. Which of the following can become resistant to antibiotics (select all that apply) –

- [ ] Bacteria
- [ ] Viruses
- [ ] Fungi
- [ ] Parasites
- [ ] Humans
- [ ] Animals

13. What are the causes of antibiotic resistance?

- [ ] poor infection prevention and control
- [ ] inadequate hand hygiene
- [ ] use of antibiotics
- [ ] overuse of antibiotics

14. Which of the following are important aspects of tackling AMR

- [ ] surveillance
- [ ] public awareness
- [ ] healthcare professional training
- [ ] infection prevention and control
- [ ] antimicrobial stewardship
- [ ] investment towards new medicines and other interventions

15. How do you dispose of antibiotics currently?

16. WHO classifies antibiotics into 3 categories in order to optimise their use and minimise the development of resistance. The three categories are
Access, Reserve and Watch. In the section below, put these categories in order of preferred use

1

2

3

17. General views about antimicrobial resistance (AMR) and antimicrobial stewardship (AMS) - all to complete

Please indicate how much you agree with the following viewpoints (1 strongly disagree, 5 = strongly agree)

	1- Strongly disagree	2- Disagree	3- Neutral	4- Agree	5- Strongly agree	I don't know
I am worried about antimicrobial resistance	○	○	○	○	○	○
Antimicrobial resistance is an important issue in my daily practice	○	○	○	○	○	○
My actions can protect the effectiveness of antimicrobials and prevent resistance	○	○	○	○	○	○
Everyone can support the public good by promoting awareness of AMR	○	○	○	○	○	○
I think prescribing guidelines support preventing antimicrobial resistance	○	○	○	○	○	○
Prescribing guidelines will be/are easy to implement in my hospital	○	○	○	○	○	○
Ensuring guideline compliance is important to prevent antimicrobial resistance	○	○	○	○	○	○

	1- Strongly disagree	2- Disagree	3- Neutral	4- Agree	5- Strongly agree	I don't know
By practising good hand hygiene/handwashing, I can support efforts to prevent the spread of infections and antibiotic resistance	◯	◯	◯	◯	◯	◯
I feel motivated to advocate for the fight against antimicrobial resistance	◯	◯	◯	◯	◯	◯
Sharing my knowledge about antimicrobial resistance can help me and my colleague's practice	◯	◯	◯	◯	◯	◯
Challenging inappropriate antimicrobial use within all healthcare staff is important in my workplace	◯	◯	◯	◯	◯	◯
When I identify inappropriate antibiotic prescribing I feel confident to highlight/challenge it with colleagues	◯	◯	◯	◯	◯	◯
Advocating for improvements in infection prevention and control is an important aspect of antimicrobial stewardship	◯	◯	◯	◯	◯	◯
Patients and the public will take antibiotics inappropriately regardless of what I do	◯	◯	◯	◯	◯	◯
It is important I give advice to patients/public about antibiotic resistance	◯	◯	◯	◯	◯	◯
Quantifying antimicrobial use is vital to identify gaps to target antimicrobial stewardship	◯	◯	◯	◯	◯	◯

	1- Strongly disagree	2- Disagree	3- Neutral	4- Agree	5- Strongly agree	I don't know
I consider antimicrobial resistance when treating a patient	◯	◯	◯	◯	◯	◯
