# Supplementary material for: Pharmacist-Led Antimicrobial Stewardship Programme in Two Tertiary Hospitals in Malawi
Source: Antibiotics (Basel). 2024 May 23;13(6):480. doi: 10.3390/antibiotics13060480 (PMC11201287; doi:10.3390/antibiotics13060480)
Supplement: Supplementary file 1 [file antibiotics-13-00480-s001.zip › Pre-Training Survey_Malawi-Wales.pdf]

Dear Colleague:

The Pharmaceutical Society of Malawi (PHASOM) and The Wales Antimicrobial Pharmacist Group (WAPG) applied for funding from the Department of Health and Social Care's Fleming fund, Commonwealth Partnership for antimicrobial stewardship (CwPAMS).

The funding application was successful, and PHASOM and WAPG agreed to develop a Health Partnership with the aim of fostering cooperation and the exchange of knowledge and skills in the areas of: antimicrobial stewardship (AMS) and antimicrobial resistance (AMR).

The purpose of the Partnership is to build professional capacity and sustainability to deliver effective antimicrobial stewardship training to healthcare and pharmacy professionals in both KCH and MCH. This will be namely the education and training of pharmacy teams regarding AMR and AMS strategies, establishing sustainable antimicrobial surveillance systems, improved antimicrobial prescribing and supporting the pharmacy teams to implement AMS strategies.
This survey will help us establish the baseline knowledge, attitudes and behaviours of pharmacists and pharmacy technicians at the KCH and MCH in regards to AMS and AMR.

We ask for the survey to be completed before your the training you are attending and then again after the training so we can understand the learning that we hope will lead to improved AMS practice, and thus lead to tangible benefits for all.

There are no right or wrong answers - you are not being assessed .
If you would like to find out more about the programme, please contact your representative in your hospital in the first instance, and for further queries (and queries directly related to this questionnaire), please get in touch at amr@commonwealthpharmacy.org.

All data collected will be stored anonymously and in line with GDPR best practice (more information can be found here: https://www.gov.uk/data-protection). There is no time limit for these questions and you are free to discontinue at any time. By completing the survey you are confirming that you have read the above and are happy to proceed.

Demographic Questions

1. Name of Partnership (FOR PILOT PHASE ONLY)

- ○ Norfolk and Suffolk NHS Foundation Trust – The Assemblies of God Hospital, Saboba
- ○ Malawi Wales Antimicrobial Pharmacist Partnership
- ○ UK Faculty of Public Health (FPH) – Ghana Public Health Association (GPHA)
- ○ North Middlesex University Hospital NHS Trust, London (NMUH) – Korle-Bu Teaching Hospital (KBTH)
- ○ University College London Hospitals NHS Foundation Trust – University of Health and Allied Sciences (UHAS), Ho
- ○ Healthcare Improvement Scotland – Ghana Police Hospital and Keta Municipal Hospital
- ○ Northumbria Healthcare NHS Foundation Trust – Kilimanjaro Christian Medical Centre (KCMC)
- ○ Cambridge University Hospitals NHS Foundation Trust – Makerere University and Mulago National Referral and Teaching Hospital
- ○ University of Salford – Pharmaceutical Society of Uganda
- ○ The University of Manchester – Gulu Regional Referral Hospital
- ○ Nottingham Trent University – Makerere University School of Public Health
- ○ London School of Hygiene and Tropical Medicine (LSHTM) – Makerere University College of Health Sciences and Infectious Diseases Research Collaboration (IDRC)
- ○ University of Sussex; Brighton and Sussex Medical School (BSMS) – University Teaching Hospital (UTH), Lusaka

2. Is your hospital a teaching hospital? (i.e. a hospital with structured teaching activities)

- ○ Yes
- ○ No

3. What is your main role in the hospital?

- ○ Medical doctor
- ○ Pharmacist
- ○ Nurse
- ○ Surgeon
- ○ Medical microbiologist
- ○ Infectious diseases specialist
- ○ Infection prevention and control (IPC) specialist
- ○ Hospital management
- ○ Other (please specify)

[ ]

4. In which department do you work?

- [ ] Medicine
- [ ] Surgery
- [ ] Paediatrics
- [ ] Obstetrics
- [ ] Accident and emergency
- [ ] Outpatient
- [ ] Other (please specify)

5. How long have you been practicing in this role?

6. We would like to be able to link your answers on this survey with answers on surveys in the future or past. Please make a code which you can enter on every survey:
- the third letter of your first name
- the month you were born (e.g., if you were born in January you would write “01”)
- the last letter of your mother’s name

Section 1: Your Views

7. Antibiotics are effective against

- [ ] Bacteria
- [ ] Viruses
- [ ] Fungi
- [ ] Parasites

8. What is antibiotic resistance?

[ ]

9. Which of the following can become resistant to antibiotics (select all that apply) –

- [ ] Bacteria
- [ ] Viruses
- [ ] Fungi
- [ ] Parasites
- [ ] Humans
- [ ] Animals

10. What are the causes of antibiotic resistance?

- [ ] poor infection prevention and control
- [ ] inadequate hand hygiene
- [ ] use of antibiotics
- [ ] overuse of antibiotics

11. Which of the following are important aspects of tackling AMR

- [ ] surveillance
- [ ] public awareness
- [ ] healthcare professional training
- [ ] infection prevention and control
- [ ] antimicrobial stewardship
- [ ] investment towards new medicines and other interventions

12. How do you dispose of antibiotics currently?

13. WHO classifies antibiotics into 3 categories in order to optimise their use and minimise the development of resistance. The three categories are
Access, Reserve and Watch. In the section below, put these categories in order of preferred use

1

2

3

14. General views about antimicrobial resistance (AMR) and antimicrobial stewardship (AMS) - all to complete

Please indicate how much you agree with the following viewpoints (1 strongly disagree, 5 = strongly agree)

	1- Strongly disagree	2- Disagree	3- Neutral	4- Agree	5- Strongly agree	I don't know
I am worried about antimicrobial resistance	○	○	○	○	○	○
Antimicrobial resistance is an important issue in my daily practice	○	○	○	○	○	○
My actions can protect the effectiveness of antimicrobials and prevent resistance	○	○	○	○	○	○
Everyone can support the public good by promoting awareness of AMR	○	○	○	○	○	○
I think prescribing guidelines support preventing antimicrobial resistance	○	○	○	○	○	○
Prescribing guidelines will be/are easy to implement in my hospital	○	○	○	○	○	○
Ensuring guideline compliance is important to prevent antimicrobial resistance	○	○	○	○	○	○

	1- Strongly disagree	2- Disagree	3- Neutral	4- Agree	5- Strongly agree	I don't know
By practising good hand hygiene/handwashing, I can support efforts to prevent the spread of infections and antibiotic resistance	○	○	○	○	○	○
I feel motivated to advocate for the fight against antimicrobial resistance	○	○	○	○	○	○
Sharing my knowledge about antimicrobial resistance can help me and my colleague’s practice	○	○	○	○	○	○
Challenging inappropriate antimicrobial use within all healthcare staff is important in my workplace	○	○	○	○	○	○
When I identify inappropriate antibiotic prescribing I feel confident to highlight/challenge it with colleagues	○	○	○	○	○	○
Advocating for improvements in infection prevention and control is an important aspect of antimicrobial stewardship	○	○	○	○	○	○
Patients and the public will take antibiotics inappropriately regardless of what I do	○	○	○	○	○	○
It is important I give advice to patients/public about antibiotic resistance	○	○	○	○	○	○
Quantifying antimicrobial use is vital to identify gaps to target antimicrobial stewardship	○	○	○	○	○	○

	1- Strongly disagree	2- Disagree	3- Neutral	4- Agree	5- Strongly agree	I don't know
I consider antimicrobial resistance when treating a patient	○	○	○	○	○	○

15. The next set of questions are grouped based on your role in the hospital. Please select an option based on your role to be taken to the next page.

- ◯ Pharmacist
- ◯ Doctor or Surgeon
- ◯ Nurse
- ◯ Other

Section 2: Antimicrobial Stewardship Practices

For Pharmacists Only

16. In the LAST MONTH how many times did you do the following?

	More than once a day	About once a day	Several times a week	About once a week	1-3 times a month	Not at all	N/A
i. I queried a prescription for an antibiotic due to insufficient evidence on the infection cause	◯	◯	◯	◯	◯	◯	◯
ii. I advised a colleague on the most appropriate antimicrobial	◯	◯	◯	◯	◯	◯	◯
iii. I safely disposed of antimicrobials at work	◯	◯	◯	◯	◯	◯	◯
iv. I contributed to the AMS strategies of my place of work	◯	◯	◯	◯	◯	◯	◯
v. I was involved in collecting data on AMR	◯	◯	◯	◯	◯	◯	◯
vi. I have followed up a patient who I have supplied an antimicrobial to	◯	◯	◯	◯	◯	◯	◯
vii. I was involved in clinical activities (give some examples here)	◯	◯	◯	◯	◯	◯	◯

I was involved in clinical activities (give some examples here)

[ ]

17. Pharmacists only

Please indicate how much you agree with the following statements (1= strongly disagree, 5 = strongly agree):

	1- Strongly disagree	2- Disagree	3- Neutral	4- Agree	5- Strongly agree	I do not understand/do not know
i. The formulation and dose I provide is often determined by stock rather than available guidelines (e.g. WHO Essential Medicines List - AWaRe recommendations or national/local guidelines)	○	○	○	○	○	○
ii. When I am concerned about a prescription I am unsure the protocol to follow to check with the prescriber	○	○	○	○	○	○
iii. When I am concerned about a prescription, the prescriber expects me to query the prescription with them	○	○	○	○	○	○
iv. When I am uncertain, I check my antibiotic choice with a peer or superior before supplying	○	○	○	○	○	○
v. I feel under pressure by patients to supply antibiotics, even when they are not clinically required	○	○	○	○	○	○
vi. I am confident in independently supplying appropriately for the treatment of infection (i.e. without prescription)	○	○	○	○	○	○
vii. I am confident in advising patients on how to use/take the antibiotic supplied	○	○	○	○	○	○

	1- Strongly disagree	2- Disagree	3- Neutral	4- Agree	5- Strongly agree	I do not understand/do not know
viii. I am aware of how antimicrobials can be safely disposed of in my place of work	○	○	○	○	○	○
ix. It is part of my role to contribute to my hospital's goal to tackle antimicrobial resistance	○	○	○	○	○	○
x. It is part of my role to collect data to support tackling AMR	○	○	○	○	○	○
xi. It is part of my role to feed back to prescribers and other colleagues about their use of antimicrobials	○	○	○	○	○	○

18. In the management of infections, which of these do you use regularly? (Select no more than 3)

- ☐ Local clinical practice guidelines
- ☐ International clinical guidelines (which?)
- ☐ Formulary
- ☐ Documentation from the pharmaceutical industry medical representatives from industry
- ☐ Previous clinical experience
- ☐ Continuing education training courses
- ☐ Infection specialists
- ☐ Scientific journals
- ☐ information from a colleague
- ☐ As directed by my senior colleague
- ☐ Professional resources/publications
- ☐ Social media
- ☐ None of the above
- ☐ I do not know
- ☐ Other (please specify)

Section 2: Antimicrobial Stewardship Practices

For any doctors and surgeons

19. Considering the last week only, roughly how often have you prescribed your patients with antibiotics?

- ◯ <10% of patients
- ◯ 10-30% of patients
- ◯ 30-50% of patients
- ◯ 50-70% of patients
- ◯ 70-90% of patients
- ◯ >90% of patients
- ◯ No antibiotics prescribed

20. What percentage of these are broad-spectrum antibiotics (target both gram-positive and gram-negative organisms or have activity against a wide range of disease causing bacteria. Examples include carbapenems (e.g meropenem), quinolones (e.g ciprofloxacin) etc)?

- ◯ <10%
- ◯ 10-30%
- ◯ 30-50%
- ◯ 50-70%
- ◯ 70-90%
- ◯ >90%
- ◯ N/A

21. In the LAST MONTH how many times did you do the following?

	More than once a day	About once a day	Several times a week	About once a week	1-3 times a month	Not at all	N/A
I provided an antibiotic due to fear of patient deterioration	◯	◯	◯	◯	◯	◯	◯
I provided multiple antimicrobials to the same patient	◯	◯	◯	◯	◯	◯	◯
I stopped an antibiotic supply earlier than the prescribed course length	◯	◯	◯	◯	◯	◯	◯
I checked my choice with a senior colleague	◯	◯	◯	◯	◯	◯	◯
I prescribed an antibiotic due to patient pressure to maintain the relationship with them	◯	◯	◯	◯	◯	◯	◯
I prescribed an antibiotic because I was uncertain about the diagnosis of infection	◯	◯	◯	◯	◯	◯	◯
I provided a broad/wider spectrum antibiotic due to doubts about which was most suitable for the patient	◯	◯	◯	◯	◯	◯	◯
I have received feedback on my antimicrobial prescribing	◯	◯	◯	◯	◯	◯	◯
I consulted prescribing guidelines prior to choosing an antimicrobial for my patient	◯	◯	◯	◯	◯	◯	◯
I have followed up a patient who I have given antimicrobials to	◯	◯	◯	◯	◯	◯	◯

22. In the management of infections, which of these do you use regularly? (Select no more than 3)

- ☐ Local clinical practice guidelines
- ☐ International clinical guidelines (which?)
- ☐ Formulary
- ☐ Documentation from the pharmaceutical industry medical representatives from industry
- ☐ Previous clinical experience
- ☐ Continuing education training courses
- ☐ Infection specialists
- ☐ Scientific journals
- ☐ information from a colleague
- ☐ As directed by my senior colleague
- ☐ Professional resources/publications
- ☐ Social media
- ☐ None of the above
- ☐ I do not know
- ☐ Other (please specify)

23. For doctors or surgeons

Please indicate how much you agree with the following statements about you and your workplace (1 = strongly disagree, 5 = strongly agree)

	1- Strong disagree	2- Disagree	3- Neutral	4- Agree	5- Strongly agree	I do not understand/do not know
I have easy access to antibiotic guidelines when I need to treat infections	○	○	○	○	○	○
I know with whom or where to access information to ensure I provide antibiotics appropriately	○	○	○	○	○	○
I can follow up patients who I have given antibiotics to	○	○	○	○	○	○
When I decide which antibiotic to use, my choice is more affected by the cost (affordability for patient) than the cause of the infection.	○	○	○	○	○	○
When I am uncertain, I check my antibiotic choice with a peer or superior before prescribing	○	○	○	○	○	○
I feel under pressure by patients to supply antibiotics, even when they are not clinically required	○	○	○	○	○	○
Fear of patient deterioration or fear of complications leads me to prescribe antibiotics even when I know they are not recommended first line	○	○	○	○	○	○
I am confident in advising colleagues on the most appropriate antimicrobial	○	○	○	○	○	○

	1- Strong disagree	2- Disagree	3- Neutral	4- Agree	5- Strongly agree	I do not understand/do not know
I am confident making antibiotic prescribing decisions in accordance with WHO essential medicines lists	○	○	○	○	○	○
I am confident making antibiotic prescribing decisions in accordance with national/local guidelines	○	○	○	○	○	○
My knowledge of antibiotics is sufficient for me to independently prescribe appropriately for the treatment of infection	○	○	○	○	○	○
I am confident in advising patients on the most appropriate antibiotic use	○	○	○	○	○	○
I expect the pharmacist to query a prescription with me if they have concerns about the prescription	○	○	○	○	○	○

Section 2: Antimicrobial Stewardship Practices

For Nurses Only

24. In the LAST MONTH how many times did you do the following?

	More than once a day	About once a day	Several times a week	About once a week	1-3 times a month	Not at all	N/A
I queried a prescription for an antibiotic due to insufficient evidence on the infection cause	○	○	○	○	○	○	○
I queried the dose of an antibiotic with the prescriber	○	○	○	○	○	○	○
I queried the dose of an antibiotic with the pharmacist	○	○	○	○	○	○	○
I administered an antibiotic for a prescription that was not in line with guidelines	○	○	○	○	○	○	○
I advised a colleague on the most appropriate antimicrobial	○	○	○	○	○	○	○
I safely disposed of antimicrobials at work	○	○	○	○	○	○	○
I contributed to the AMS strategies of my place of work	○	○	○	○	○	○	○
I was involved in collecting data on AMR	○	○	○	○	○	○	○
I have followed up a patient who I have given antimicrobial to	○	○	○	○	○	○	○
I was involved in clinical activities (give some examples here)	○	○	○	○	○	○	○

I was involved in clinical activities (give some examples here)

[ ]

25. Nurses only

Please indicate how much you agree with the following statements (1 = strongly disagree, 5 = strongly agree):

	1- Strongly disagree	2- Disagree	3- Neutral	4- Agree	5- Strongly agree	I do not understand/do not know
The formulation and dose I administer is often determined by stock rather than AWaRe recommendations	○	○	○	○	○	○
When I am concerned about a prescription I am unsure the protocol to follow to check with the prescriber	○	○	○	○	○	○
When I am concerned about a prescription, the prescriber expects me to query the prescription with them	○	○	○	○	○	○
When I am uncertain, I check my antibiotic choice with a peer or superior before administering	○	○	○	○	○	○
I feel under pressure by patients to administer antibiotics, even when they are not clinically required	○	○	○	○	○	○
I am confident in independently prescribing appropriately for the treatment of infection	○	○	○	○	○	○
I am confident in advising patients on the most appropriate antibiotic use	○	○	○	○	○	○
I understand how antimicrobials can be safely disposed of in my place of work	○	○	○	○	○	○

	1- Strongly disagree	2- Disagree	3- Neutral	4- Agree	5- Strongly agree	I do not understand/do not know
It is part of my role to contribute to my hospital's AMR strategy	◯	◯	◯	◯	◯	◯
It is part of my role to collect data on AMR	◯	◯	◯	◯	◯	◯
It is part of my role to feed back to prescribers and other colleagues about their use of antimicrobials	◯	◯	◯	◯	◯	◯

26. Collecting data on hospital-wide antimicrobial use through Global PPS will support antimicrobial stewardship through in the institution: (List three ways)

1

2

3

Section 3 - Awareness of AMS and IPC Interventions

27. What initiatives are you aware of in your organisation which focus on antibiotic awareness and resistance?

- [ ] Infection guidelines
- [ ] Antibiotic formulary
- [ ] Awareness campaigns
- [ ] Other (please specify)

28. Have you heard of World Antibiotic Awareness Week (WAAW)?

- ○ Yes
- ○ No
- ○ Unsure

29. Have you participated in World Antibiotic Awareness Week (WAAW)? (e.g. led an awareness campaign, shared posts on social media about antibiotic awareness)

- ○ Yes
- ○ No
- ○ Unsure
