# Supplementary material for: Pharmacist-Led Antimicrobial Stewardship Programme in Two Tertiary Hospitals in Malawi
Source: Antibiotics (Basel). 2024 May 23;13(6):480. doi: 10.3390/antibiotics13060480 (PMC11201287; doi:10.3390/antibiotics13060480)
Supplement: Supplementary file 1 [file antibiotics-13-00480-s001.zip › S1 Training Timetable.pdf]

Malawi/Wales Antimicrobial Pharmacist Partnership - AMS training plan

Time	Activity	Training points covered	Lead	Resources needed
8am<br>-<br>8.30am	Welcome and sign in, networking and introductions<br>Introduce timetable and aims for day<br><br>Videos patient experience running in background as participants arriving<br>Patient experience - For Malawi 15Apr2022 - YouTube	Understanding patient experience of AMR, pharmacist role	Aggrey<br>Training lead	Training slides,<br>Computer<br>Projector<br>Screen<br>Routers for WiFi<br>Sign in sheet with permissions<br>Copies of toolkit<br>Name labels
8.30am<br>-<br>9am	Complete pre-training questionnaire<br><br>Ice breaker<br>Introduce yourself, pharmacy role and say something others will not know about you	Understanding of pre-training knowledge<br><br>Networking for participants	Training lead, Aggrey	Ppaper copies of questionnaire
9am<br>- 10am	Part 1: Introduction to antimicrobial resistance	AMR introduction<br>What is AMR, how does is develop and spread,<br>Global an national action plans<br>Extent of AMR issue in Malawi<br>Lab upgrade and Fleming fund work	Training lead	See above
10am -<br>10.15am	Tea break			
10.15am<br>–<br>11.15pm	Part 2 – All about antibiotics	Understanding clinical infection<br>Antibiotics mechanism of action<br>Good antibiotic prescribing practice<br>Empirical v prophylaxis v colonisation<br>IV/PO switches<br>Antibiotic Culture & Sensitivity	Training lead	As per part 1
11.15pm<br>-<br>12.15pm	Case studies<br>A. CAP<br>B. UTI	Understanding of infection management and clinical pharmacy role	Training lead to introduce<br>2 facilitators<br>2 or 4 groups	Copies of case studies

	Small group work and feedback 15mins to answer questions, 5 minutes to feedback to wider group		depending on numbers	
12.15pm - 1.15pm	Lunch break			
1.15pm - 2.15pm	Part 3 - Antimicrobial Stewardship and putting this into action	Understanding of AMS and the role of the pharmacy team<br>AMS tools	Training lead	As per part 1 & 2
2.15pm - 2.45pm	AMS game	Understanding of AMS and AMR learning	Training lead	2 copies of AMS game
2.45pm – 3pm	Tea Break			
3pm - 3.45pm	Small group work AMS in action<br>• Choose an antibiotic intervention<br>• Plan the intervention<br>◦ What tools will you use?<br>◦ Where will you implement the intervention<br>◦ Who will be involved?<br>◦ Timeframe?<br>• Feedback to group on your AMS intervention	AMS implementation and how to do this	Facilitator per group	
3.45pm - 4pm	Closing remarks and questions		Training lead	
