# Supplementary material for: Pharmacist-Led Antimicrobial Stewardship Programme in Two Tertiary Hospitals in Malawi
Source: Antibiotics (Basel). 2024 May 23;13(6):480. doi: 10.3390/antibiotics13060480 (PMC11201287; doi:10.3390/antibiotics13060480)
Supplement: Supplementary file 1 [file antibiotics-13-00480-s001.zip › S2 Malawi-Wales Antimicrobial Pharmacy Partnership Antimicrobial Stewardship Toolkit outline.pdf]

S2: Malawi-Wales Antimicrobial Pharmacy Partnership Antimicrobial Stewardship Toolkit outline

- Antimicrobial resistance (AMR)
  - Resistance rates in Malawi & the national response
  - Impact of resistance
- Antimicrobial stewardship (AMS)
  - Benefits of AMS
  - WHO AWaRe classification
- Preventing infection
  - Vaccination
  - Infection Prevention and Control
  - Antimicrobial Prophylaxis
- The general approach to infection management
- Determining the need for treatment
  - Does the patient have an infection?
  - Self-limiting infections
  - Watchful waiting
  - Sepsis
  - Taking cultures
  - Interpreting previous culture results
- Initial choice of therapy
  - Choice of antibiotic
  - Spectrum of cover
  - Route
  - Malawi Standard Treatment Guidelines
  - Allergy
    - Assessing penicillin 'allergy'
    - Risk of cross-sensitivity
  - Timing
  - Documentation
- Optimising dosage
  - Renal impairment
  - Hepatic impairment
  - Body weight
  - Drug interactions
  - TB drug monographs
  - Myasthenia gravis
  - Monitoring for adverse Reactions
  - Administration
  - Pregnancy and Lactation
  - Children
- Review of therapy by 72 hours
  - Targeting therapy
    - Interpreting culture results
  - IV to oral switch (IVOS)
  - Duration
- Patient education & public engagement
- Further resources
- Appendix 1: Flowchart for Infection Management
- Appendix 2: Usual bacterial causes of infection
- Appendix 3: Spectrum of activity of common antibiotics
- Appendix 4: Clinical Pharmacy Resources
- Appendix 5: Snapshot audit template
